# Mechanical Properties Evaluation of Polymer-Binding C-S-H Structure from Nanoscale to Macroscale: Hydroxyl-Terminated Polydimethylsiloxane (PDMS) Modified C-S-H

**DOI:** 10.3390/ma15238361

**Published:** 2022-11-24

**Authors:** Zheyu Zhu, Yue Zhou, Zhishan Huang, Zhongping Wang, Yuting Chen

**Affiliations:** 1 Key Laboratory of Advanced Civil Engineering Materials of Ministry of Education, School of Materials Science and Engineering, Tongji University, Shanghai 201804, China; 2 Civil Engineering, Harbin Institute of Technology, Harbin 150001, China

**Keywords:** calcium silicate hydrate, mechanical properties, PDMS, AFM–FM, molecular dynamics simulation

## Abstract

Exploring and modifying the C-S-H structure at a micro–nano level is an effective solution to improve the performance of Portland cement. Compared with organics inserting C-S-H, the research on the performance of a polymer-binding C-S-H structure from nanoscale to macroscale is limited. In this work, the mechanical properties of a modified C-S-H, using hydroxyl-terminated polydimethylsiloxane (PDMS) as the binders, are evaluated. The PDMS-modified C-S-H structures are introduced into macro-defect-free cement to obtain stress–strain curves changes at a macro scale. The AFM–FM was adopted to measure the morphology and elastic modulus of C-S-H at a nano scale. The molecular dynamics (MD) simulation was performed to assess the toughness, tensile properties, and failure mechanism. The results show that the PDMS-modified C-S-H powders change the break process and enhance ductility of MDF cement. The elastic modulus of PDMS-modified C-S-H is lower than pure C-S-H. When PDMS molecules are located between the stacking crystal units, it can enhance the toughness of C-S-H aggregates. The PDMS-modified C-S-H stacking structure has better plasticity, and its tensile strains are higher than the pure C-S-H. PDMS molecules hinder the initial crack expansion, leading to the branching of the initial crack. In addition, the measurement of AFM–FM can identify and obtain the mechanical properties of basic units of C-S-H. This paper enhances the understanding of cement strength sources and modification methods.

## 1. Introduction

Calcium silicate hydrate (C-S-H) is the dominate material of Portland cement, which determines the overall properties of final structures [1,2,3,4,5]. Therefore, modifying the micro–nanostructure of C-S-H is one effective solution to enhance and functionalize the construction materials. Comparing with the study on the basic units of C-S-H, the stacking characteristics of C-S-H units attracted the attention of most scholars. Jennings proposed the CM-I and CM-II models to describe the stacking structure of C-S-H units [6,7]. The CM-I describes the least structural unit in C-S-H gels sized at 5.6 nm [7]. The CM-II describes these stacked units as nanoscale crystals [6]. Researchers believe that C-S-H stacking units are similar to layered natural minerals [8,9,10,11,12,13,14], such as tobermorite and jennite, detailed structures of which can be seen in Ref [15,16]. The widely investigated C-S-H structure provides a solid foundation for the structure and properties modification.

Natural organisms mostly use flexible substances to make up for the defects of inorganic materials. Inspired by this, researchers tried to modify the performance of cementitious materials by preparing organic-modified C-S-H at atomic level. Using tobermorite-like crystals, Matsuyuma [17,18] proposed the insertion of polymers into a C-S-H structure for the first time. Subsequent studies have shown that various polymers have been used to insert in the layer of C-S-H, such as polyvinyl alcohol (PVA) [19], polyethylene glycol (PEG) [20,21], polyacrylamide (PAM) [22], polyaniline (PAN) [23,24,25], polyacrylic acid (PAA) [26], and protein [27]. Some scholars demonstrate that at nanoscale, inserting polymers into C-S-H structures produces more superior properties than the classical C-S-H obtained in the hydrates [23,24].

For polymer/C-S-H system, the other possible structure is that polymer may bind C-S-H stacking units instead of through insertion [19,28,29,30]. As the results in our previous study show [28,29,30], when forming polymer-binding C-S-H, the polymers can react with C-S-H by covalent bonding and hydrogen bonding. For example, Zhu [29] demonstrated that PDMS locates between adjacent C-S-H stacking particles, which modified both the chemical structure and stacking mode. PDMS molecules (i) coat on the C-S-H surface by forming films; and (ii) exist and link the adjacent C-S-H particles through Si–O–Si covalent bond. Another polymer, such as pre-gelatinized starch, can form organic-binding C-S-H structures through hydrogen bonding. The performance of C-S-H can be enhanced by the above polymer-modified C-S-H method. For example, the polymer-inserted C-S-H structures have better anti-corrosion of salts, indicating a better biologically stable structure, in theory.

No matter how the polymer/C-S-H structure mentioned above is successfully designed in a synthetic process, the method to realize polymer/C-S-H structures in real Portland cement is still limited. One solution is to introduce synthetic polymer/C-S-H particles into Portland cement hydration. However, the introduction method, mechanical properties from nanoscale to macroscale, and failure process need further research.

Since the pores have a great influence on the mechanical properties, the macroscale polymer/C-S-H effects should be evaluated in the cement products that eliminate most pores. Among this, the macro-defect-free (MDF) material is suitable. MDF is a highly dense composite material. The preparation of MDF materials includes three steps: (i) mixing cement (CAC, Portland, gypsum etc.) with a small amount of polymers and water; (ii) high shearing for eliminating porosity; and (iii) pressurized processing under a certain temperature for molding and curing. Since no cracks larger than 150 μm exist in the MDF cement, one of MDF’s advantages in performance is high tensile strength, which successful achieves more than 150 Mpa. Another advantage in performance of MDF is in electrical, magnetic, and acoustic properties, and low temperature, which is determined by the adopted polymer types and composites nanostructure. When the Portland cement is adopted to prepared MDF, pressurized processing plays a significant role in eliminating porosity and the main hydrate is C-S-H. Considering that MDF materials almost eliminate the pores effects, introducing the special C-S-H powders into MDF cements benefits organic/C-S-H properties and create special MDF Portland cement.

This paper takes C-S-H modified by PDMS as the target to evaluate the mechanical properties of polymer-binding C-S-H structures. At a macro scale, the PDMS/C-S-H particles are introduced into macro-defect-free cement to obtain stress–strain curve changes. At a nano scale, the stacking morphology, mechanical properties, and C-S-H basic stacking blocks were firstly determined by AFM–FM, and the molecular dynamics simulation was adopted to evaluate the toughness and failure process of PDMS-modified C-S-H structures. The PDMS-bridged effects, which bind adjacent stacking C-S-H nanoparticles, and the initial cracks propagation can be revealed.

## 2. Experimental

### 2.1. Materials and Samples

According to the previous studies [29], the samples used for C-S-H modification were prepared in the following way: solution A consisting of a mixture of ethyl orthosilicate (TEOS), anhydrous ethanol (EtOH), DL-tartaric acid, calcium nitrate, and deionized water was stirred at 50 °C for 20 min. The molar ratio of 1:0.83:2:0.02:1 was obtained among TEOS, calcium nitrate, deionized water, DL-tartaric acid, and EtOH. Subsequently, PDMS was added to solution A at 50 °C while stirring, which, upon completion, changes into a yellow sol. The 2 g sol was then added into the 10 g NaOH (4 mol/L) solution under stirring at 50 °C for 2 h. The acquired samples were maintained by water bath treatment at 60 °C for 7 d. Finally, the obtained 1.5 g white precipitate (modified C-S-H powders) was washed with 1000 mL deionized water four times, and was dried at 50 °C. In this paper, the samples used for analysis are consistent with previous studies, and partial information of XRD, FITR, NMR, TG-DTG, and SEM are suggested in Ref. [29]. The pure, original C-S-H is labelled as PDMCSH00, and the modified C-S-H is labelled as PDMCSH50.

The macro-defect-free (MDF) sample were prepared as follows: the Portland cement was mixed with PDMCSH00 and PDMCSH50 powders. Then the powders were molding into 4 × 7.5 × 32 mm under 5 MPa to obtain hardened blocks (three samples for one group). The hardened blocks were immersed into water and cured at 50 °C for 7 d. Finally, the samples were dried at 50 °C.

### 2.2. Characterization Methods

#### 2.2.1. Stress and Strain Testing of MDF Cement

According to GB/T 6569-2006 of China, the samples are tested by three points bending method. The distance between two fulcrums is 30 mm. The rate of stroke is 0.0001 mm/s. The stainless steel standard sample are used for calibration. The flexural stress (*σ*) and strain (*ε*) is calculated as follows:

$$\sigma =\frac{1.5\times f\times l}{b\times h^{2}}\;\varepsilon =\frac{6\times h}{l^{2}}\times d$$

where *f* is failure load when subjected to flexure (N); *l* is the distance between two fulcrums (mm); *b* is width of specimen section (mm); *h* height of specimen section (mm); *d* is stroke of testing pressure head.

#### 2.2.2. AFM–FM Measurement

The dried PDMCSH50 powders were tested for their mechanical properties at 25 °C, and the test was performed under nitrogen to avoid water in the air. The instrument Asylum MFP-3D (Bruker, Karlsruhe, Germany), made in the USA, was adopted for the measurement. It is usually used to measure the mechanical properties of materials at the nanoscale. It can control test environments such as liquid or air. For the target microregion, the amount of data collected can meet the requirements of data analysis. The dried powders were directly glued onto the surface film composed of polyethylene. The in situ measurement of the elastic modulus and morphology were then obtained under the AM–FM mode. The procedures for the MFP-3D AFM were discussed in Ref [31,32,33,34]. For the FM mode, calculation of the elastic modulus (*E*) can be described by the following equation:

$$E=\alpha \sqrt{\frac{8}{RA_{1}}}\times \frac{k_{2\;}^{2}f_{01}}{k_{1}f_{02}^{2}}\times \frac{\Delta f_{2}^{2}}{\Delta f_{1}}$$

where *k*_1_ and *k*_2_ represent the probe spring constants of the first and second modes, respectively; *A*_1_ describes the first bending frequency of instantaneous amplitude; α is automatically obtained by comparing the calibration and test samples; *R* denotes the radius of probe tip; and *f*_01_ and *f*_02_ are the first and second bending frequencies of probe oscillations, respectively. Here, mica and the AC200 probe (Olympus, Bruker, Billerica, MA, USA) were used for calibration and all measurements, respectively.

#### 2.2.3. AFM–FM Measurement

Beginning with material preparation, the 4 mm^3^ cube of samples were mixed with a low viscosity epoxy resin. The epoxy was subsequently cured, and the specimen was then cut into 1 cm^3^ with the cutting machine, ground and polished to achieve 7 mm thick samples with parallel surfaces. For viewing cement samples, typical settings are an accelerating voltage of 12 kV and probe currents of 2 nA.

#### 2.2.4. Molecular Dynamics Simulation

##### Model Construction

**Figure 1:**
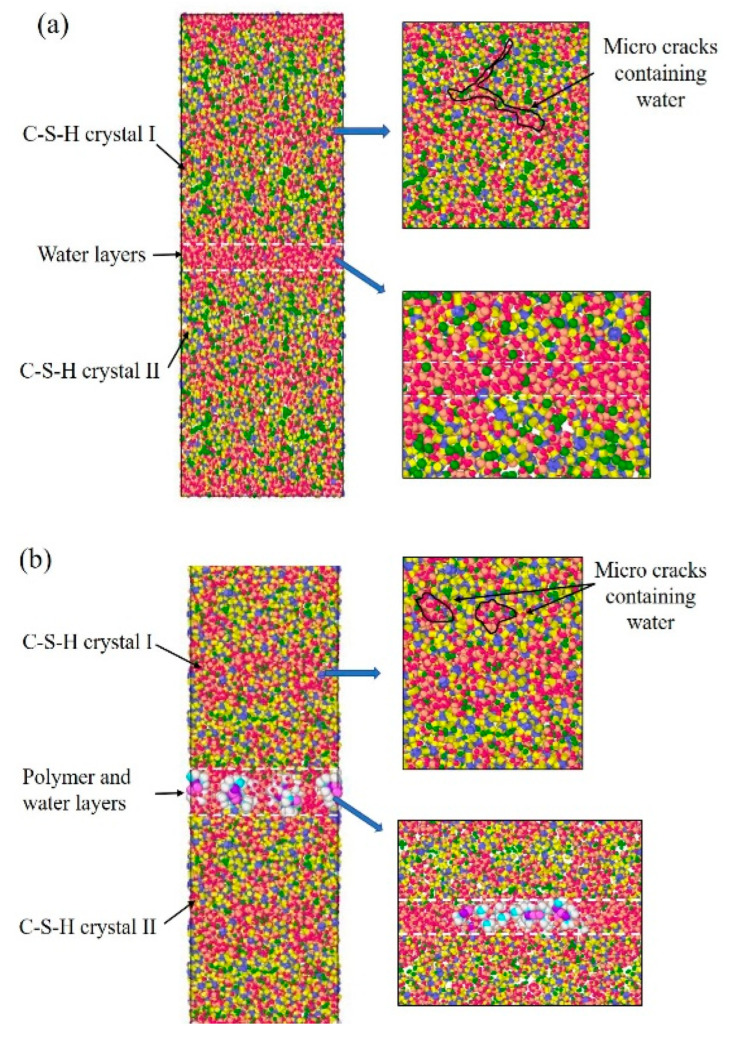
The stacking model of C-S-H: (**a**) pure C-S-H; (**b**) PDMS-modified C-S-H.

C-S-H crystals were constructed using 11Å-tobermorillonite as the initial structure (Figure 1). First, the dry cells of 11Å-tobermorillonite were established. Subsequently, the ratio of calcium to silica (Ca/Si) was adjusted to 1.1 by removing the silicon–oxygen tetrahedra and calcium ions. The Q*^n^* distribution was satisfied with the ^29^Si NMR study results. The content of Q^0^, Q^1^, and Q^2^ were set to 13%, 67%, and 20%, respectively. Water molecules (298 K, 1 g/cm^3^) were added by grand canonical Monte Carlo (GCMC) method until reaching water saturation state. The dimensions of the C-S-H cell are 48 × 40 × 57 Å^3^. Subsequently, the above two C-S-H cells were stacked along the *z*-axis and the parallel-aligned PDMS molecules were stacked in the middle of the two C-S-H cells. Considering the previous experimental results, the Si-O-R groups of the PDMS molecule were connected to the silicon–oxygen tetrahedra of C-S-H by Si–O–Si bonds. Finally, the middle layer containing organic molecules was saturated with water molecules (300 K, 1 g/cm^3^) by GCMC. Energy optimal calculation of PDMS-modified C-S-H model was performed using ClayFF and CVFF dual force fields. The NVT system was used to balance 1 ns (0.1 fs per step). The model developed contained 8700 to 8900 atoms. The number of atoms involved in the model and the equilibrium time ensured the accuracy of the mechanistic analysis data. Theoretically, the weak region in the C-S-H structure will fracture first when subjected to the force. In this experiment, several dry cracks were randomly constructed inside the C-S-H crystal, and the GCMC placed the cracks in a water-saturated state. The mechanically weak regions are the middle layer between the C-S-H stacking particles and the water-containing cracks within the C-S-H crystals.

##### Uniaxial Stretching Test

The optimal energy calculation (300 K) of the proposed model was performed using NPT synthesis to release the excess stresses, ensuring there was no external pressure in the *x*, *y*, and z directions. The PDMS-modified C-S-H model was stretched along the *x*, *y*, and *z* axes at a rate of 0.08 ps^−1^. When the model is stretched along a certain axis, the forces in the other two directions, which are orthogonal to the principal stress, are zero. The value of the pressure in the tensile direction is equal to the internal pressure.

Assessing the toughness of materials should consider the stress and strain changes. Here, the toughness change of PDMS-modified C-S-H is assessed by strain energy per unit volume. The strain energy per unit volume is the potential energy stored within the material by the formation of stress and strain. The formula for calculating the strain energy per unit volume is:

En=∫σ d ε

where *En* is the strain energy per unit volume; *σ* is the stress; ε is the strain.

## 3. Results and Discussion

### 3.1. Evaluation of Mechanical Properties at Macro Scale

#### 3.1.1. The Application of PDMS-Modified C-S-H in MDF Cement

**Figure 2:**
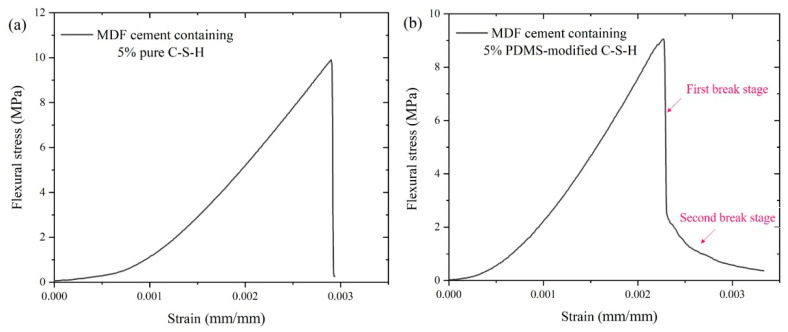
Flexural strength–strain curves for MDF cement containing: (**a**) 5% PDMCSH00; (**b**) 5% PDMCSH50.

Figure 2 is the flexural stress–strain curves for MDF cement. In Figure 2a, the maximum flexural stress is 9.8 MPa. When achieving the maximum value of stress, the sample breaks immediately, and the plastic deformation stage does not appear in MDF cement containing 5% PDMCSH00. This indicates that the pure C-S-H does not change the fracture process of MDF cement, which is consistent with the results of inorganic materials. In Figure 2b, when adding 5% PDMCSH50, the stress–strain curves change, in which the break process can be divided into two stages. At the first stage, the stress reduces rapidly, and then at the second stage, the stress decreases slowly. The maximum flexural stress is 9.3 MPa, which is almost consistent with MDF cement containing 5% pure C-S-H. It is noteworthy that when adding 5% PDMCSH50, the strain increases 13.7% more than in MDF cement containing 5% PDMCSH00. Thus, the PDMCSH50 powders change both the break process and ductility of MDF cement. In addition, during the elastic deformation stage, the elastic moduli in Figure 2a,b are 23.0 MPa and 20.8 MPa, respectively. This indicates that the PDMCSH50 is a flexible particle. In other words, the PDMCSH50 particles may act as flexible fibers in enhancing the MDF cements, and the second break stage is due to the flexibility of PDMCSH50.

#### 3.1.2. BSE Image of MDF Cement

**Figure 3:**
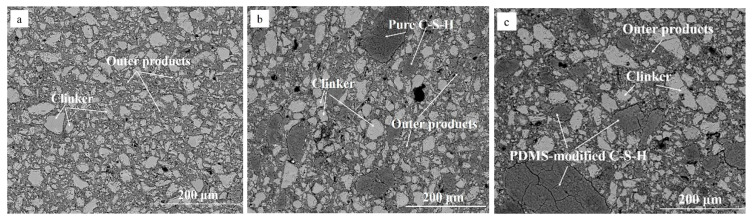
BSE images of MDF cement containing: (**a**) 0% C-S-H powders; (**b**) 5% PDMCSH00; (**c**) 5% PDMCSH50.

To evaluate the microstructure of MDF cement containing PDMCSH50 particles, the BSE image is measured. Figure 3 is BSE image of MDF cement. In Figure 3a, the MDF cement sample without adding C-S-H powders contains unhydrated clinker (white region) and outer products (grey regions). This structure is similar with hydrated Portland cements, and the pressure process make the structure more compacted. In Figure 3b,c, when adding C-S-H powders, the microstructure of MDF cement changes, in which pure C-S-H (PDMCSH00) particles and PDMS-modified C-S-H (PDMCSH50) particles are bound by outer products. Comparing with unhydrated clinker, both the PDMCSH00 particles and the PDMCSH50 particles are larger. Such artificially introduced C-S-H regions can contribute to the mechanical properties of MDF cement. Thus, the break stage change discussed above is due to the properties of those two large C-S-H regions. If the large C-S-H region is flexible, it may act as a fiber in connecting the mixture structure. In addition, in Figure 3c, during bending more cracks appear in PDMS-modified C-S-H particles, which may lead to a higher ductility by consuming more energy. That is, the flexible C-S-H region contributes a new mechanics characteristic of MDF cement. To evaluate the tensile properties of PDMCSH00 and PDMCSH50 at nanoscale, the AFM–FM and molecular dynamics (MD) simulation are preformed below.

### 3.2. Evaluation of Mechanical Properties at Nanoscale

#### 3.2.1. Morphology and Elastic Modulus

**Figure 4:**
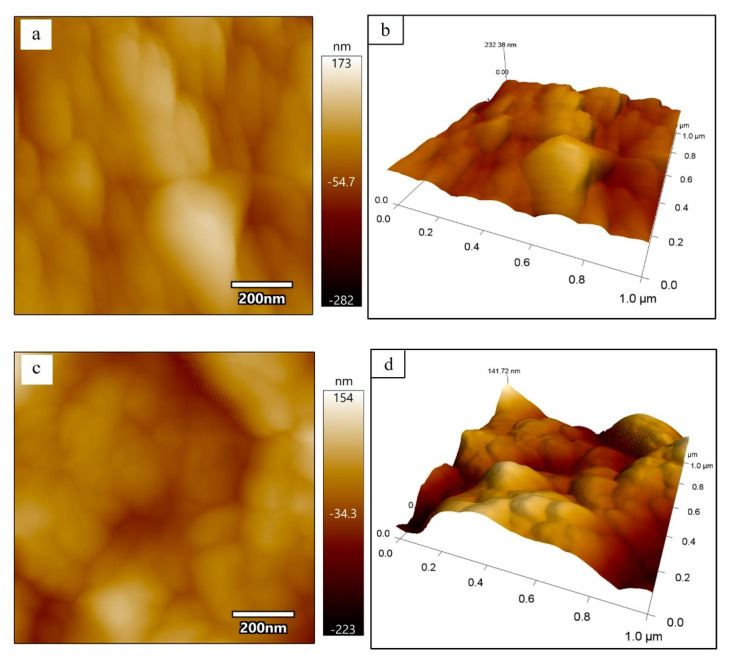
Morphology of (**a**) and (**b**) PDMCSH00; (**c**) and (**d**) PDMCSH50.

Figure 4 is the morphology of PDMCSH00 and PDMCSH50. In Figure 4a,b, the PDMCSH00 shows a stacking of spherical particles with diameter ranges from 50 nm to 200 nm. In Figure 4c,d, PDMCSH50 is a stacking of C-S-H spherical particles with diameter ranges from 30 nm to 150 nm. The PDMS does not change the shape of C-S-H nanoparticles, and the sizes of PDMCSH50 are smaller than that of pure C-S-H. According to the CM-I model, C-S-H nanoparticles are the aggregates of 5 nm basic units [6]. In this study, no 5 nm sized C-S-H units are observed by AFM image. When the C-S-H nanoparticles are less than 100 nm, it is difficult to obtain satisfactory images with the AFM test. This is consistent with the recent research [35]. Despite this, the information of 5 nm units is obtained by elastic modulus, which is discussed below. Since the sample preparation is treated without grinding, the nanostructure here is the true surface of C-S-H particles.

**Figure 5:**
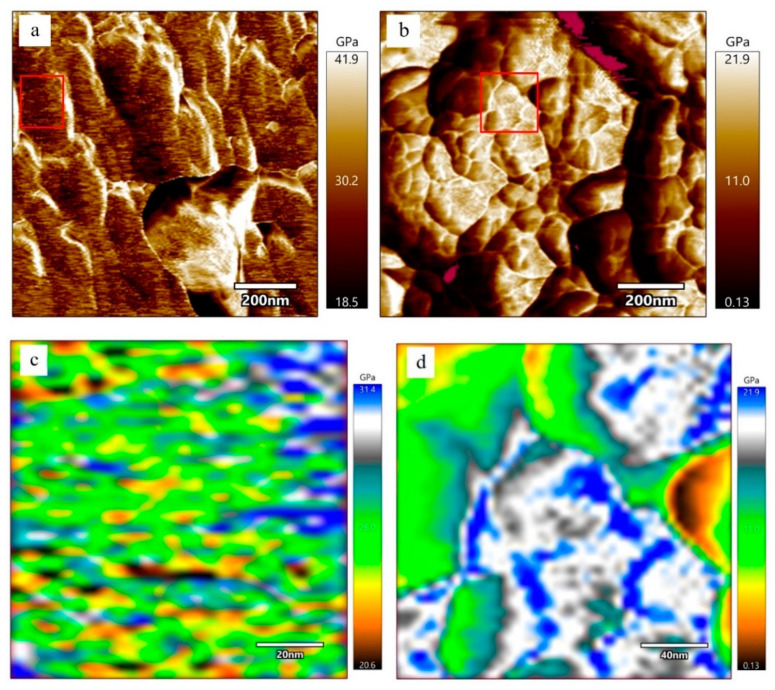
Elastic modulus spatial distribution of stacking units of pure C-S-H and PDMS-modified C-S-H at nanoscale: (**a**) and (**c**) pure C-S-H; (**b**) and (**d**) PDMCSH50.

Figure 5 is the elastic modulus distributions of PDMCSH00 and PDMCSH50 samples. AFM–FM can measure both the elastic modulus and morphology information at the same location simultaneously. In Figure 5a, the elastic modulus values of pure C-S-H nanoparticles are unevenly distributed, and the average elastic modulus of pure C-S-H is 30.2 GPa. This value is similar to the C-S-H nanoparticles measured in the existing literature [35]. Figure 5b is the elastic modulus distribution of the PDMCSH50 sample. In Figure 5b, the elastic modulus of PDMCSH50 is also unevenly distributed, and the average elastic modulus of PDMCSH50 is 10.9 GPa. The elastic modulus of PDMS-modified C-S-H is lower than that of pure C-S-H. Compared with pure C-S-H, the average elastic modulus of PDMCSH50 is reduced by about 63.9%. Jennings suggests that the large C-S-H particles are formed by the aggregates of nano-stacking basic units. Such basic units can be globules around 5 nm as described in the CM-I model, or nanocrystals (≤10 nm) as described in the CM-II model [6,35]. The red rectangular area in Figure 5a,b was selected to evaluate the elastic modulus of C-S-H basic units.

In Figure 5c, the elastic modulus of pure C-S-H basic units is about 20.6 GPa to 31.4 GPa. In detail, the yellow and brown basic units are 20.0 GPa~24.0 GPa, which are spherical nanoparticles with a size range from 5 nm 10 nm. The green and blue basic units are 26.0 GPa~32.0 GPa, which form a layered arrangement of nanowires. There are two explanations for the uneven elastic modulus distribution of basic units. According to the CM-I model, the 5 nm globules have different compositions and microstructures. According to the CM-II model, the other explanation involves the spatial orientation of crystals, composition, and microstructure. Thus, nanowires in Figure 5c are formed either by colloids with similar structure and composition, or by nanocrystals with the same orientation and arranged direction. The yellow and brown areas are colloid or nanocrystals with different structures and compositions.

In Figure 5d, the elastic modulus of PDMCSH50 ranges from 0.13 GPa to 21.9 GPa. The yellow, green, and white regions are evenly distributed, and none of yellow or green nanowires or globules can be observed. This is due to PDMS linking adjacent stacking units, leading to the formation of larger regions with similar mechanical properties. Since the elastic modulus of pure PDMS film is about 2.0~50.0 MPa [36], the yellow, green, and white areas are mixed structures of PDMS and C-S-H. The elastic modulus of the mixed structures is determined by the content of PDMS and stacking units. It is still noteworthy that the blue areas are nanowires that are formed by connected spherical stacking units. In a word, PDMS changes both the stacking mode and elastic modulus.

#### 3.2.2. Probability Density Distributions of Elastic Modulus

**Figure 6:**
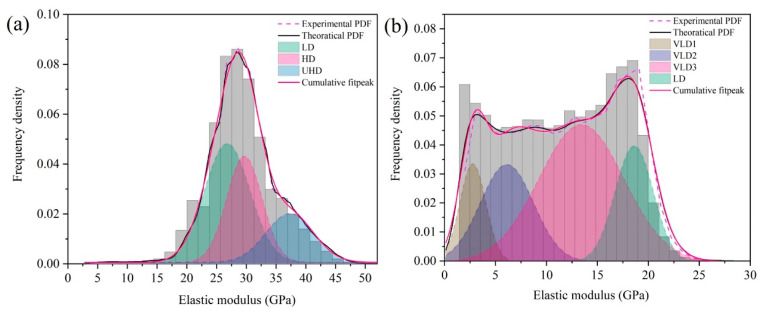
Probability density distributions of elastic modulus of: (**a**) PDMCSH00; (**b**) PDMCSH50.

According to the different elastic modulus, C-S-H can be divided into very-low-density C-S-H (VLD), low-density C-S-H (LD), high-density C-S-H (HD), and ultra-high-density C-S-H (UHD) phases [35,37]. In Portland cement hydrates, the elastic modulus of LD, HD, and UHD is about 21.7 ± 2.2 GPa, 29.4 ± 2.4 GPa, and 45.0 GPa, respectively [38,39,40]. Zhu [35] et al. demonstrate that the elastic modulus of VLD is about 11.0 GPa. In order to quantify the elastic modulus of PDMS-modified C-S-H, the values obtained in Figure 6a,b were statistically analyzed according to the kernel density estimation model (KDE) [35]. Then the Gaussian equation was further used to deconvolve the theoretical PDF, and the area of the deconvolution peak was the relative content of different C-S-H.

Figure 6 is the probability density distributions of the PDMCSH00 and PDMCSH50 elastic moduli. In Figure 6a,b, the PDMCSH00 consists of LD, HD, and UHD. PDMCSH50 consists of VLD and LD. No HD and UHD exists on the surface of the PDMCSH50 samples. Table 1 is probability density distributions of the elastic moduli of PDMCSH00 and PDMCSH50. In Table 1, for PDMCSH00, the stacking units of LD, HD, and UHD are 47.0%, 32.8%, and 20.2%, respectively; for PDMCSH50, the stacking units of VLD and LD are 81.5% and 18.5%, respectively, indicating that the stacking units of PDMS-modified C-S-H are dominated by VLD. In addition, the VLD of PDMCSH50 samples can be deconvolved to VLD1 (2.8 GPa), VLD2 (6.1 GPa), and VLD3 (13.4 GPa), and their contents are 10.7%, 21.8%, and 49.0%, respectively.

Zhu [35] demonstrated that gel pores can be formed when nano-C-S-H is stacked. This would lead to a reduction in the elastic modulus. In this experiment, the penetration depth of the AFM tip in the *z*-axis direction is only hundreds of picometers. Thus, the elastic modulus value represents the characteristics of the C-S-H stacking unit. The modulus of PDMS film is about 2.0~50.0 MPa [36]. If the thickness of PDMS is larger than the depth of *z*-axis compression, the test value should be 2.0~50.0 MPa, because the AFM tip only penetrate on the PDMS film. However, the elastic moduli in this experiment are much higher than that of PDMS film, which indicates that the testing values represent the mechanical properties of the PDMS/C-S-H hybrid structure. In addition, the different elastic modulus of VLD1, VLD2, and VLD3 may be caused by the variation in PDMS in the mixed structures. With the PDMS increases, the elastic modulus decreases, which indicates that PDMS-modified C-S-H is more prone to deformation.

### 3.3. Evaluation of Mechanical Properties by Molecular Dynamics Simulation

#### 3.3.1. Tensile Properties

**Figure 7:**
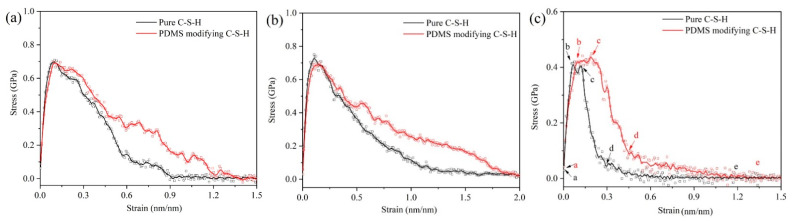
Tensile stress–strain curves for C-S-H and PDMS-modified C-S-H: along the (**a**) *x*-axis; (**b**) *y*-axis; (**c**) *z*-axis.

Figure 7 shows the tensile stress–strain curves of pure C-S-H and organic-modified C-S-H. For all the stretching direction in Figure 7, the PDMS-modified C-S-H structure shows better plasticity and strain variables than pure C-S-H. In Figure 7a,b, when the stretching is along the x and y axes, the stress–strain curves of the PDMS-modified C-S-H and pure C-S-H almost coincide in the elastic stage. After reaching the yield point, for PDMS-modified C-S-H, the stress decline becomes slower. This indicates that when PDMS participates in C-S-H stacking structures, the maximum tensile stress does not increase, but the ductility is improved. Recent simulations show that along the x and y axes, the stress of C-S-H crystals without cracks is about 1.0 to 2.0 GPa [41,42]. In our research, the stress along the x and y axes is about 0.65~0.75 GPa, which is due to the fact that the C-S-H crystals, water-containing cracks, and interlayer structures all contribute to the stress–strain curve.

The stretching along the *z*-axis represents the separation of two C-S-H particles [43]. In Figure 7c, when the loading stretches along the *z*-axis, the maximum stress of pure C-S-H and PDMS-modified C-S-H is less than that along the x and y axes. It is noteworthy that the stress–strain curve along the *z*-axis shows a yield stage (b to c in Figure 7c). Compared with pure C-S-H, the yield stage of PDMS-modified C-S-H is longer. When the stretching is along the *z*-axis, the direction of the force is perpendicular to the middle layer, thus, the yield stage is related to the failure process of middle layer. After the yield point, the stress decline of PDMS-modified C-S-H is slower than that of pure C-S-H, which indicates that PDMS can increase the ductility. The previous study shows that the elastic modulus of PDMS-modified C-S-H is smaller and more prone to deformation. Here, the molecular dynamics simulation shows that PDMS-modified C-S-H structure has better plasticity, which is consistent with the experimental conclusion.

#### 3.3.2. Toughness

**Figure 8:**
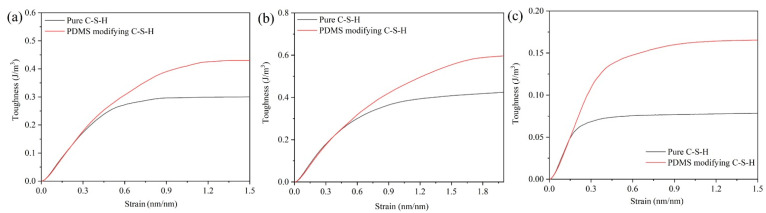
Volumetric energy of and C-S-H and PDMS-modified C-S-H along the (**a**) *x*-axis; (**b**) *y*-axis; (**c**) *z*-axis.

Figure 8 is the strain energy per unit volume (*En*) of pure C-S-H and PDMS-modified C-S-H. In Figure 8, the *En* value of PDMS-modified C-S-H is higher than that of pure C-S-H, which indicates that PDMS-modified C-S-H has better toughness. When stretching along the *x*-axis, the *En* value of pure C-S-H and PDMS-modified C-S-H is 0.30 J/m^3^ and 0.43 J/m^3^, respectively. When the stretching is along the *y*-axis, the *En* value of pure C-S-H and PDMS-modified C-S-H is 0.42 J/m^3^ and 0.60 J/m^3^, respectively. When the stretching is along the *z*-axis, the *En* value of pure C-S-H and PDMS-modified C-S-H is 0.07 J/m^3^ and 0.16 J/m^3^, respectively. Compared with pure C-S-H, when the stretching is along *x*, *y*, and *z* axes, the *En* value of PDMS-modified C-S-H increases by 43.3%, 42.8%, and 128.6%, respectively. It is noteworthy that the *En* values of pure C-S-H and PDMS-modified C-S-H coincide in the elastic stage, which indicates that the toughness is similar in the elastic deformation stage. In addition, compared with pure C-S-H, the PDMS-modified C-S-H structure has better toughness in the plastic deformation stage.

#### 3.3.3. Toughening and Failure Mechanism

As mentioned above, stretching along the *z*-axis represents the separation process of adjacent stacking particles. In this process, the water-containing cracks and the mechanical weak areas in the inner C-S-H crystal leads to the disintegration of the structure. The structural changes of pure C-S-H and PDMS-modified C-S-H can be divided into four stages: (i) initial structure, (ii) elastic deformation, (iii) plastic deformation, and (iv) structural separation.

**Figure 9:**
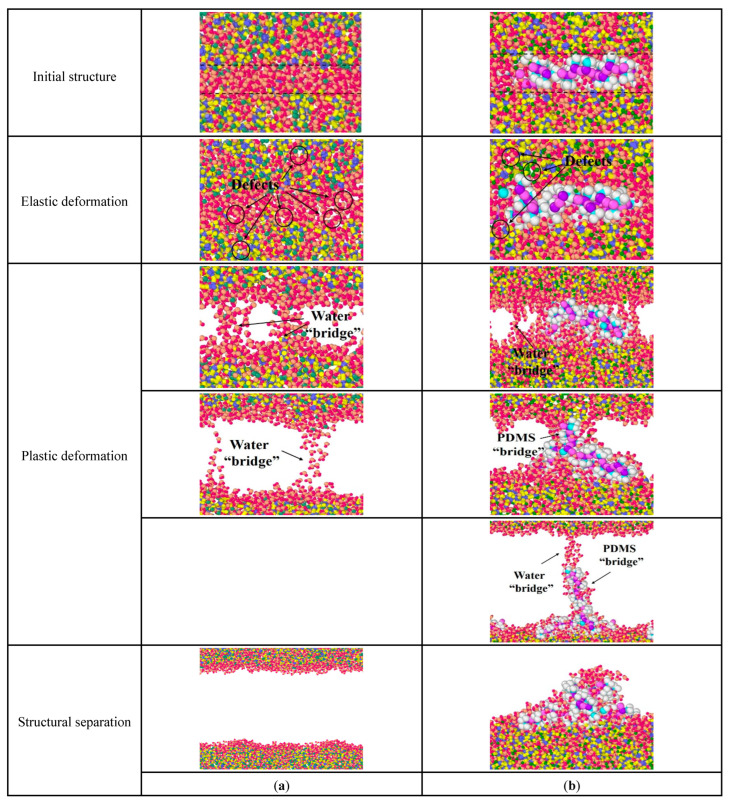
Structural changes of C-S-H stretched along the *z*-axis: (**a**) pure C-S-H; (**b**) PDMS-modified C-S-H.

Figure 9a is the structural changes of pure C-S-H stretched along the *z*-axis. When stretched along the *z*-axis, the extension of the initial crack in the water molecular layer is the main factor separating the C-S-H stacking structure. In Figure 9a, for pure C-S-H, a small number of atomic defects appear in both the water molecular layer and the C-S-H crystal during the elastic deformation. This indicates that both the water molecular layer and the C-S-H crystal undergo structural changes. During the plastic deformation, the initial cracks form in the water molecular layer and gradually expand. Then the C-S-H crystals are only connected by the water molecular bridge. When the water molecular bridge breaks, the stacking structure of C-S-H crystals is completely separated.

Figure 9b is the structural changes of PDMS-modified C-S-H stretching along the *z*-axis. In Figure 9b, for PDMS-modified C-S-H, during elastic deformation small amounts of atomic defects appear in the middle layer and inside the C-S-H crystal, which the shape of the PDMS molecules changes to offset the energy of the stretching process. During the plastic deformation, the initial crack first appears in the middle layer and then expands, which forms a water molecule bridge. As the stretching continues, the water molecule bridge breaks and then PDMS molecules connect C-S-H to pile up particles, which form a PDMS molecular bridge. When the PDMS molecular bridge breaks, the water molecule bridge forms again along the stretching direction of PDMS molecules. Then, the water molecule bridge breaks again, and the stacking C-S-H crystals completely separate. Finally, the PDMS molecules “fall back” and adsorb on the surface of the C-S-H crystal. Compared with pure C-S-H, the stretching mode of PDMS molecules changes in the elastic and plastic deformation. PDMS increases the required energy to destroy the C-S-H stacking structure, and then leads to better toughness.

**Figure 10:**
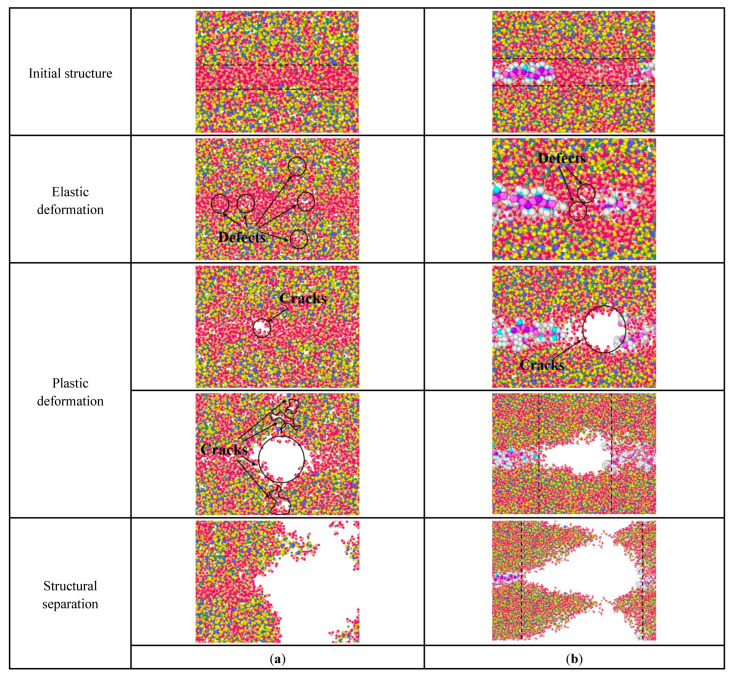
Structural changes of C-S-H stretched along the *x*-axis: (**a**) pure C-S-H; (**b**) PDMS-modified C-S-H.

When the stretching is along the *x*-axis, the direction of the force is parallel to the stretch direction of the PDMS molecule. Figure 10 is the internal structural changes of C-S-H stretched along the *x*-axis. In Figure 10a, during the elastic deformation, when pure C-S-H is stretched, atomic defects are generated firstly, to adapt to the energy change. Then, in the plastic deformation, the initial cracks first appear in the water molecular layer and gradually expand, leading to form independent cracks groups, which are arranged perpendicular to the tensile direction. When these initial crack groups are connected and expand into a large size crack, the pure C-S-H stacking structure separates.

In Figure 10b, the failure process of PDMS-modified C-S-H is also related to the initial crack development. During the elastic deformation, atomic defects first appear in the water molecular layer. During the plastic deformation, these initial cracks gradually expand and eventually lead to the failure of stacking structure. It is noteworthy that there are some micro-regions containing only water molecules in the middle layers, since PDMS molecules cannot be extended infinitely. Then, the initial crack expands mainly in this microregion, leading to the failure of the PDMS-modified C-S-H structure. In other words, when the tensile force is parallel to the tensile direction of PDMS molecules, the microregion containing PDMS molecules has a more stable structure. As the initial crack expansion breaks more silicon–oxygen tetrahedral chains, more energy is expended, leading to increased toughness. In sum, PDMS tends to break more silicone tetrahedral chains by prolonging the expansion path of cracks, which consumes more energy.

**Figure 11:**
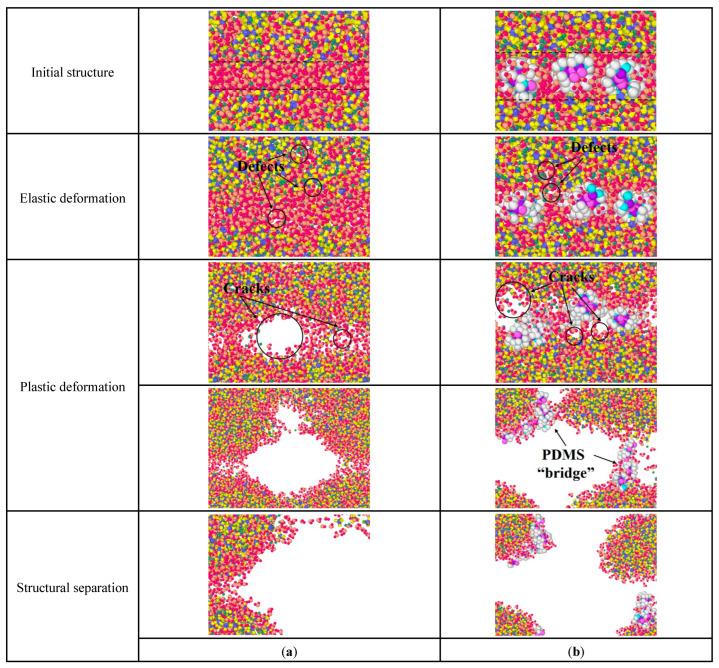
Structural changes of C-S-H stretching along the *y*-axis: (**a**) pure C-S-H; (**b**) PDMS-modified C-S-H.

Figure 11 is the structural changes of C-S-H stretching along the *y*-axis. In Figure 11a, pure C-S-H produces some atomic defects during the elastic deformation. During the plastic deformation, the initial cracks appear first in the middle layer and then expand along the water-containing cracks in the C-S-H crystal until fracture. In comparison, for the PDMS-modified C-S-H structure, the initial crack expansion path changes. In Figure 11b, during the plastic deformation, the initial crack expands along the water-containing cracks in the middle layer and C-S-H crystals, forming C-S-H fragments that are connected by the PDMS molecular bridge. When the PDMS molecular bridge is broken, the PDMS-modified C-S-H structure fails. That is, PDMS molecules can hinder the expansion of initial cracks, and make the initial crack tend to bypass the PDMS molecules. This increases the length of the initial crack and, thus, consumes more energy, which leads to increased toughness.

In summary, the initial cracks in the C-S-H stacking structure first generate in the water molecular layer. Subsequently, these initial cracks continue to grow and expand along the water-containing cracks inside the C-S-H crystal, eventually leading to structural failure. PDMS has the effect of hindering crack expansion and forming molecular bridges, increasing the energy required for crack expansion, which is macroscopically manifested as an increase in toughness. Considering the fact that C-S-H gels are not perfect crystals, a reasonable water-containing crack is beneficial to enhance the toughness of C-S-H gels.

## 4. Conclusions

Calcium silicate hydrate highly determines the properties of Portland cement. Compared with organics inserted into C-S-H, the research on the performance of organic-binding C-S-H nanostructures is still limited. This paper explores the mechanical properties of PDMS-modified C-S-H stacking structures at various scales. The PDMS-modified C-S-H structures are introduced into macro-defect-free cement to obtain stress–strain curves change at macro scale. The elasticity modulus is measured experimentally by AFM–FM. The toughness, tensile properties, and failure mechanism are evaluated by molecular dynamics simulation.

When PDMS molecules are located between the stacking units, the PDMS improves the toughness of the C-S-H aggregates. The PDMS-modified C-S-H are dominated by ultra-low-density stacking units with an average elastic modulus of 10.9 GPa, which is about 60% lower than that of pure C-S-H. The PDMS-modified C-S-H stacking structure has better plasticity, and its tensile strains are higher than the pure C-S-H. Compared with pure C-S-H, when stretching along the *x*, *y*, and *z* axes, the PDMS-binding C-S-H stacking structure increases strain energy per unit volume by 43.3%, 42.8%, and 128.6%, respectively. The initial cracks first generate in the water molecular layer, and the crack expansion leads to structural failure. The PDMS molecules hinder the initial crack expansion, leading to the branching of the initial crack. When added in MDF cement, the PDMS-modified C-S-H powders changes the break process and enhance the ductility of MDF cement. This paper enhances the understanding of the source of strength, and the method to improve the toughness, of cement materials.

## Figures and Tables

**Table 1:** Probability density distributions of elastic modulus of PDMCSH00 and PDMCSH50.

Stacking Unit	C-S-H		PDMCSH50	
	Elastic Modulus/GPa	Content/%	Elastic Modulus/GPa	Content/%
VLD1 (%)	-	-	2.8	10.7
VLD2 (%)	-	-	6.1	21.8
VLD3 (%)	-	-	13.4	49.0
LD (%)	26.8	47.0	18.6	18.5
HD	29.7	32.8	-	-
UHD	37.3	20.2	-	-
